# National and regional challenges of antibiotic prescribing patterns in pediatric populations across Africa: a systematic review and meta-analysis

**DOI:** 10.1186/s13643-026-03223-9

**Published:** 2026-06-10

**Authors:** Sancho Pedro Xavier, Ana Raquel Ernesto Manuel Gotine, Melsequisete Daniel Vasco, Nerys Wendy Antonieta Alfane, Nelson Domingos Cote, Audêncio Victor

**Affiliations:** 1 School of Public Health, University of São Paulo (USP), Avenida Doutor Arnaldo, 715, São Paulo, São Paulo 01246904 Brazil; 2 Institute of Collective Health, Federal University of Bahia, R. Basílio da Gama S/N, Canela, Salvador, BA 40110-040 Brazil; 3 Faculty of Medicine of Bahia, Federal University of Bahia, Rua Dr. Augusto Viana, S/N - Canela, Salvador, BA 40301-155 Brazil; 4 Instituto Gonçalo Moniz, Fundação Oswaldo Cruz, Rua Waldemar Falcão, 121, Candeal, Salvador, Bahia 40296-710 Brazil; 5 Faculty of Epidemiology and Population Health, London School of Hygiene & Tropical Medicine, Keppel Street, London, WC1E 7HT UK

**Keywords:** Antibiotic prescribing, Pediatrics, Antimicrobial resistance, AWaRe, Africa, Systematic review, Meta-analysis

## Abstract

**Background:**

Excessive and inappropriate use of antibiotics represents a major concern directly associated with the emergence of resistant microorganisms, posing a global public health threat. Inappropriate prescribing patterns are particularly frequent in pediatric populations, leading to significant clinical and epidemiological consequences. Improving the quality of antibiotic prescribing is a rational and necessary strategy to strengthen the AWaRe framework (Access, Watch, and Reserve), proposed by the World Health Organization (WHO) to guide rational antibiotic use. This study aimed to systematically review and conduct a meta-analysis on antibiotic prescribing patterns in pediatric patients across Africa.

**Methods:**

We systematically searched PubMed, Embase, SciElo, Scopus, Web of Science, and the WHO Global Index Medicus on 23 May 2025. Eligible studies reported antibiotic prescribing patterns among pediatric patients in Africa, including types of prescribed antibiotics, route of administration, and AWaRe classification. Methodological quality was assessed using the National Institutes of Health (NIH) tools. Pooled prevalence estimates were calculated using random-effects meta-analysis based on the Freeman–Tukey double arcsine transformation.

**Results:**

The pooled prevalence of antibiotic prescribing among pediatric patients in Africa was 76.0% (95% CI, 68.0-82.0; *I*^2^ = 99.8%). Approximately 78.0% received at least one antibiotic parenterally, with a mean of 1.55 antibiotics per patient (95% CI, 1.36-1.74). The highest prescribing prevalence was observed in Central Africa (85.0%), followed by Western (83.0%), while Eastern and Southern regions reported a prevalence of 64.0%. By AWaRe classification, Access antibiotics accounted for 65.0% of prescriptions, Watch for 27.0%, and Reserve for 3.0% (reported only in South Africa). The most prescribed antibiotics included amoxicillin (54.1%), amikacin (47.5%), gentamicin (41.2%), and ampicillin (38.0%) in the Access group; ceftriaxone (48.3%), cefuroxime (42.0%), and imipenem/meropenem (32.9%) in the Watch group; and ampicillin combined with cloxacillin (28.0%) in the not-recommended group.

**Conclusions:**

These findings provide a descriptive overview of antibiotic prescribing patterns among pediatric patients in Africa. Given the variability across studies, the results underscore the importance of strengthening antimicrobial stewardship initiatives and promoting the rational use of antibiotics, particularly within the AWaRe framework, to support efforts aimed at mitigating antimicrobial resistance.

**Supplementary Information:**

The online version contains supplementary material available at 10.1186/s13643-026-03223-9.

## Introduction

Antimicrobial resistance (AMR) is one of the leading global public health threats, directly associated with the excessive and inappropriate use of antibiotics, which fosters the emergence and dissemination of resistant microorganisms [1]. This issue is particularly frequent in pediatric patients [2, 3], due to the high burden of infectious diseases in this age group and the challenges in early diagnosis, factors that often result in empirical antibiotic prescribing [4]. Global estimates indicate that AMR is responsible for approximately 700,000 deaths annually, disproportionately affecting low- and middle-income countries (LMICs), where the uncontrolled growth of resistance poses a major challenge [5]. In 2019, an estimated 1.20 million deaths were directly attributable and 4.94 million associated with AMR; in 2021, these figures slightly decreased to 1.14 million and 4.71 million, respectively [6]. In the same year, for the African region, 1.05 million deaths were associated with bacterial resistance, of which about 250,000 were directly attributable [7]. Projections suggest that by 2050, AMR could be linked to as many as 10 million deaths globally [8].

In Africa, the high prevalence of infectious diseases combined with fragile health systems increases reliance on empirical antibiotic prescribing, thereby favoring overuse [9]. Evidence suggests that improving infection prevention and control programs in LMICs healthcare settings could prevent at least 337,000 AMR-associated deaths annually equivalent to saving 923 lives per day, or one every two minutes [9]. The impact is particularly severe in Africa, especially in sub-Saharan Africa, which bears a disproportionate burden of antimicrobial-resistant healthcare-associated infections worldwide [10, 11]. In pediatric patients, the situation is especially critical, as children under five are more vulnerable to respiratory and diarrheal infections, often treated with antibiotics even when viral in origin [12]. Excessive and inappropriate prescribing is among the key drivers of resistance, with up to 50% of all antibiotic prescriptions in humans considered unnecessary, and at least half of treatments deemed inappropriate [13].

Recent studies indicate that antibiotic consumption in children across Africa is high [14, 15], with ceftriaxone, metronidazole, penicillin, and gentamicin being the most commonly prescribed antibiotics [16]. In this context, the implementation and strengthening of antimicrobial stewardship programs are essential, as they can reduce AMR by promoting rational and sustainable prescribing practices [17]. One of the tools recommended by the World Health Organization (WHO) is the AWaRe classification, which organizes antibiotics into three groups: Access, which includes narrow-spectrum antibiotics considered first- and second-line choices for empirical treatment of common infections and should be widely available; Watch, which encompasses broader-spectrum antibiotics with a higher risk of inducing resistance; and Reserve, which refers to last-resort antibiotics [18]. The AWaRe model is therefore an essential strategy to tackle AMR, promoting rational and safe antibiotic use across different healthcare contexts [19].

Several studies conducted in African countries have examined antibiotic prescribing patterns in pediatric patients, some of which applied the AWaRe framework. However, to date, no study has systematically and quantitatively synthesized these estimates in an integrated manner, considering the application of this tool. By consolidating the available evidence, this systematic review and meta-analysis contributes to global efforts led by the WHO, the Sustainable Development Goals (SDGs), and the One Health initiative to address AMR, often referred to as a “silent pandemic.” This study aims to fill this gap by providing a comprehensive synthesis of the existing literature on challenges in antibiotic prescribing among pediatric patients across different regions of Africa.

## Methods

### Study protocol

The identification of records, title and abstract screening, and full-text eligibility assessment for inclusion in the final analysis were conducted in accordance with the PRISMA (Preferred Reporting Items for Systematic Reviews and Meta-Analyses) guidelines [20]. This study was registered in PROSPERO under the reference number CRD42025637627, available at: https://www.crd.york.ac.uk/PROSPERO/view/CRD42025637627.

### Search strategy

Eligible studies were identified through a systematic search of PubMed, Embase, SciElo, Scopus, Web of Science, and WHO Global Index Medicus databases, with no restrictions on language or publication date. The search strategy combined the terms “Pediatric,” “Paediatric,” “Children,” “Neonates,” and “Infants” with “Antibiotic prescribing patterns,” “Antibiotic use,” “Antibiotic prescription,” and “Use of antibiotics,” along with the keyword “Africa” (S1. Table 1). The search was conducted on May 23, 2025.

### Study selection

Two members of the research team (NWAA and MVD) independently screened all titles and abstracts after the initial removal of duplicates, using the Rayyan platform. Observational studies (cross-sectional and cohort) conducted in African countries that assessed children ≤ 18 years of age, focusing on antibiotic prescription in hospital, community, or outpatient settings, were included. The outcomes of interest were: antibiotic prescribing patterns, compliance with international guidelines (AWaRe classification), and the most frequently prescribed antibiotics. Studies conducted outside the African context or not involving paediatric populations were excluded. Two members of the research team (MVD and NWA) independently reviewed and assessed the full texts of potentially eligible articles, and any disagreements were resolved by a third reviewer (SPX) through discussion until consensus was achieved.

### Study outcome

The outcome of interest was antibiotic prescription. Prescribing patterns were defined by the distribution of antibiotic prescriptions and by the World Health Organization’s AWaRe classification (Access, Watch, Reserve, unclassified, and not recommended categories).

### Data extraction and risk of bias assessment

Data from eligible studies were independently extracted by two reviewers (NDC and SPX) into an Excel spreadsheet template suggested in a previous study [21]. Each entry was subsequently verified by the principal investigator to ensure accuracy and consistency. The extracted data included: author, year of publication, country, African region, study design, population characteristics, patient type, AWaRe classification, absolute numbers for calculating antibiotic consumption, and types of antibiotics prescribed.

The methodological quality of included studies was assessed using the NIH Quality Assessment Tool for Observational Cohort and Cross-Sectional Studies. This tool comprises 14 questions designed to evaluate study quality, with categorical responses (Yes (1), No (0), or Other (CD, NR, NA), where CD indicates “cannot determine”; NA, “not applicable”; and NR, “not reported”). The overall score was calculated by summing the scores across all items, with “yes” scored as one and “no” or “NA” scored as zero. Each study was then classified as poor (0-5 points), fair (6-9 points), or good (10-14 points) [21].

### Data analysis

Proportions were pooled using random-effects meta-analysis models with the Freeman-Tukey double arcsine transformation [22]. The random-effects approach was chosen to account for the substantial methodological and contextual variability among the included studies, including differences in populations, settings, and study protocols. Fixed-effect models assume a common underlying effect across studies, which would be inappropriate in this context given the observed heterogeneity [23].

**Fig. 1:**
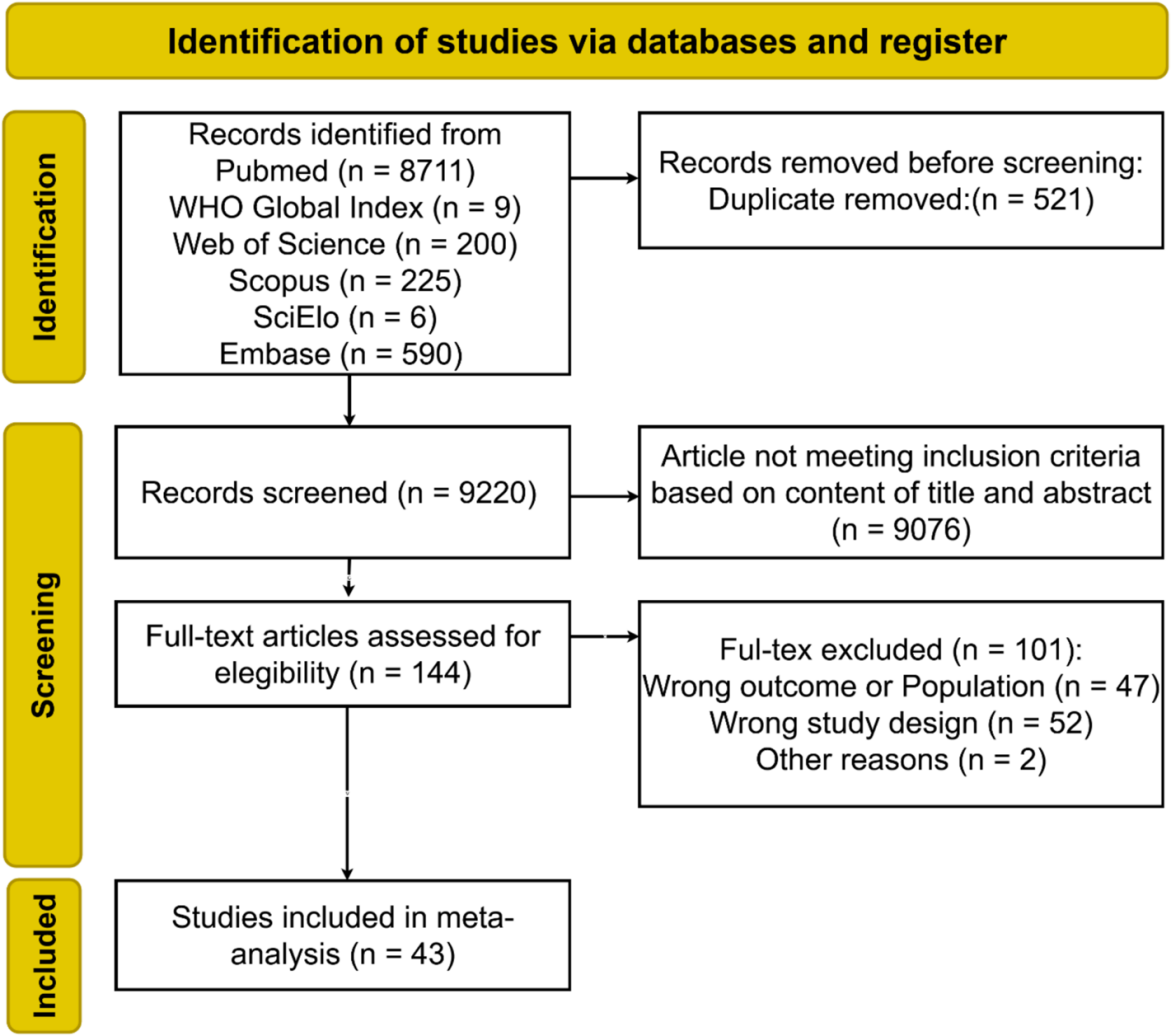
PRISMA flow diagram illustrating the study selection and screening process

To calculate the raw mean (MRAW), data on the average number of antibiotics prescribed per patient were extracted from the included studies. Considering the high heterogeneity, sensitivity analyses were conducted using the DerSimonian-Laird random-effects method [23], which incorporates between-study variability. Although the DerSimonian-Laird estimator can produce narrower confidence intervals when heterogeneity is extreme or the number of studies is small, it is widely accepted for exploratory synthesis and provides a useful check on the robustness of pooled prevalence estimates. The results of these sensitivity analyses are presented in the Supplementary Material (S3. Figure 1–24).

Quantitative AWaRe synthesis was restricted to studies explicitly reporting aggregated counts or proportions of Access, Watch, and Reserve antibiotics. Studies without primary AWaRe reporting had antibiotics classified according to the WHO AWaRe framework for descriptive purposes only and were not included in the pooled analyses, as antibiotic- or subgroup-level derivations may violate independence assumptions and reduce interpretability. AWaRe proportions were calculated using antibiotic-level denominators, defined as the number of antibiotics in each AWaRe category divided by the total number of antibiotics reported in each study.

Heterogeneity was assessed using the *I*^2^ statistic [24] and Cochran’s *Q* test [25]. The *I*^2^ index reflects the proportion of observed variance, with values of 25%, 50%, and 75% or higher considered to indicate low, moderate, and high heterogeneity, respectively [26]. Publication bias was assessed through visual inspection of funnel plot symmetry, followed by Egger’s test [27] and Begg’s rank test [28]. A *p*-value < 0.05 was considered indicative of publication bias. Where publication bias was suspected, the Trim and Fill method was applied to estimate the number of potentially missing studies and adjust the pooled effect size by imputing hypothetical studies to achieve funnel plot symmetry, providing a more robust and less biased overall estimate [29].

Subgroup analyses were conducted according to geographic region (Western Africa, Central Africa, Eastern Africa, and Southern Africa), study design (cohort and cross-sectional), patient type (inpatient, outpatient, or both), sample size (< 200, 200–500, > 500), study period (≤ 2014, 2015-2019, and ≥ 2020), and study quality (poor, fair, and good). Since subgroup analyses did not fully explain the observed heterogeneity, univariable meta-regression models were initially applied to explore potential moderators of the pooled estimates. Variables with at least one category presenting a *p*-value < 0.25 in the univariable analyses were subsequently included in a multivariable meta-regression model [30]. Additionally, a leave-one-out sensitivity analysis was performed by sequentially removing one study at a time to evaluate the influence of individual studies on the overall pooled estimates [31].

## Results

### Characteristics of included studies

**Fig. 2:**
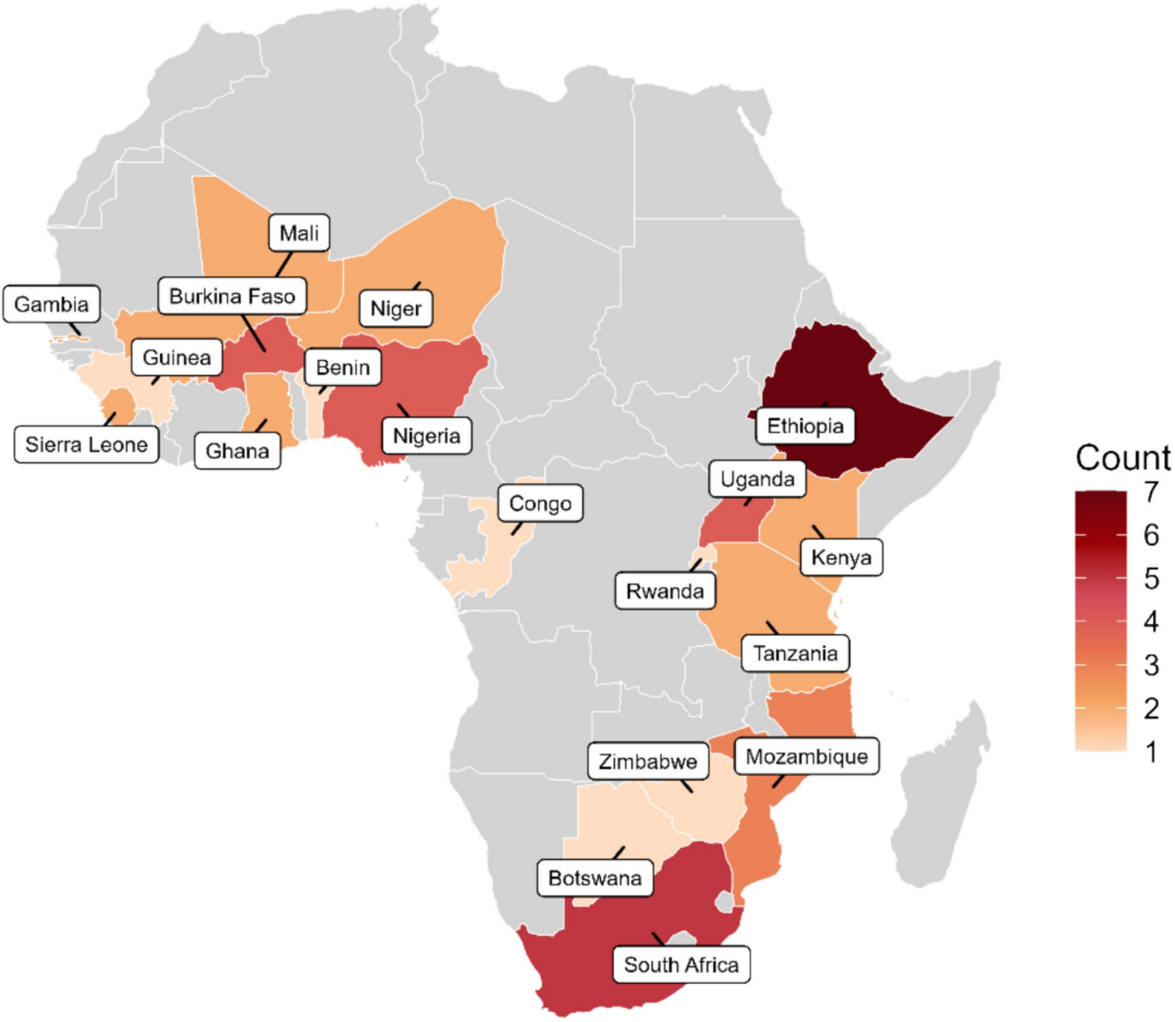
Distribution of the countries included in the studies

After removing duplicates and excluding ineligible records, 43 articles were included (Fig. 1), encompassing 61 individual studies, as some articles reported data from more than one country (Table 1). Altogether, these studies included 118,996 participants and reported 66,744 antibiotic prescription events in paediatric populations, which were incorporated into the meta-analysis. The studies were identified from PubMed, Embase, SciElo, Scopus, Web of Science, and the WHO Global Index Medicus. Although no date restriction was applied in the search strategy, the included studies covered the period 2010-2024. All studies included in this systematic review were conducted in Sub-Saharan Africa. Western Africa included Nigeria, Ghana, Benin, Burkina Faso, Senegal, Sierra Leone, The Gambia, Mali, and Guinea; Eastern Africa included Ethiopia, Kenya, Tanzania, Uganda, Rwanda, and Mozambique; Central Africa included the Democratic Republic of Congo; and Southern Africa included South Africa, Botswana, and Zimbabwe (Fig. 2; Tables 1 and 2). The majority (*n* = 38) were cross-sectional studies, with the remaining five being cohort, and sample sizes ranged from fewer than 200 to over 500 participants. Both inpatient and outpatient populations were represented. Regarding methodological quality, based on the NIH assessment, 4.65% (2/43) of the included articles were rated as poor quality (S2. Table 2).

**Table 1:** Characteristics of studies included in the systematic review and meta-analysis

**Basic information**						**Study design and eligibility criteria**			**Population of study, sample size & outcome**		
**Author, Year**	**Publication year**	Study period*	**Journal**	**Country**	**Region of Africa**	**Study Design**	**Exclusion criteria**	**Type of patients**	**Subgroups**	**Sample size**	**Outcome**
Umeokonkwo et al., 2023 [32]	2023	2017, 2018, 2019	Journal of Infection Prevention	Nigeria	Western Africa	Cross-sectional	Children admitted after 8.00 am on the day of the survey	Inpatient	Not reported	191	Antibiotic prescribing
Tack et al., 2023 [33]	2023	2022	The American journal of tropical medicine and hygiene	Democratic Republic of Congo	Central Africa	Cohort	Not specified	Inpatient	> 28 days and < 5 years	1867	Antibiotic prescribing
Tekleab et al., 2017 [34]	2017	2016	Pediatric Health, Medicine and Therapeutics	Ethiopia	Eastern Africa	Cross-sectional	Children of age 2–59 months who had diarrhea and/or cough with comorbid illness	Outpatient	2 to 59 months (≤ 5 years)	1073	Antibiotic prescribing
Okoye et al., 2022 [35]	2022	2022	BMJ Paediatrics Open	Nigeria	Western Africa	Cross-sectional	Illegible prescriptions	Outpatient	< 12 years	800	Antibiotic prescribing
Cole et al., 2015 [36]	2015	2013	Journal of Basic and Clinical Pharmacy	Sierra Leone	Western Africa	Cross-sectional	Prescriptions outside the study period, for inpatients, and for patients over 5 years of age	Outpatient	< 5 years	294	Antibiotic prescribing
Sana et al., 2019 [37]	2019	2016	Pan African Medical Journal	Burkina Faso	Western Africa	Cross-sectional	Not specified	Outpatient	≤ 5 years	1206	Antibiotic prescribing
Slogrove et al., 2010 [38]	2010	2006	Southern African Journal of Epidemiology and Infection	South Africa	Southern Africa	Cohort	Not specified	Inpatient	Neonates	195	Antibiotic prescribing
Salau et al., 2023 [39]	2023	2016, 2019	Pan African Medical Journal	South Africa	Southern Africa	Cross-sectional	Not specified	Inpatient	< 6 years	336	Antibiotic prescribing
Chaw et al., 2018 [40]	2018	2015	Antimicrobial Resistance & Infection Control	Gambia	Western Africa	Cross-sectional	Patients discharged against medical advice and records with missing or lost antibiotic/diagnosis data	Inpatient	> 28 days to 15 years	917	Antibiotic prescribing
Awuor et al., 2023 [41]	2023	2016	*Clinical Infectious Diseases*	Gambia, Mali and Kenya	Western Africa Kenya (Eastern Africa)	Cross-sectional	Infants < 2 months or with WHO-indicated antibiotic use by clinical diagnosis	Outpatient	0–59 months	4840	Antibiotic prescribing
Kamara et al., 2024 [42]	2024	2022	Antimicrobial Resistance & Infection Control	Sierra Leone	Western Africa	Cross-sectional	Outpatient and emergency pediatric records; patients admitted after 8:00 a.m.; topical or ophthalmic antibiotic use	Inpatient	≤ 17 years	150	Antibiotic prescribing AWaRe group
Olaru et al., 2020 [43]	2020	2018	Journal of Chemotherapy	Zimbabwe	Southern Africa	Cohort	Did not specify	Inpatient	≥ 5 years	130	Antibiotic prescribing
Kitt et al., 2022 [44]	2022	2018	SAGE Open Medicine	Botswana	Southern Africa	Cohort	Neonates as they are admitted to the neonatal unit	Inpatient	< 13 years	299	Antibiotic prescribing
Merga et al., 2024 [45]	2024	2022	Antimicrobial Stewardship & Healthcare Epidemiology	Ethiopia	Eastern Africa	Cross-sectional	Outpatients discharged before 8:00 a.m. but still hospitalized; neonates of uncertain or nonviable status	Inpatient	14 years	132	Antibiotic prescribing
Akintan et al., 2024 [46]	2024	2015, 2017, 2018, 2019	BMC Pediatrics	Nigeria	Western Africa	Cross-sectional	Did not specify	Inpatient	< 18 years	260	Antibiotic prescribing AWaRe group
Obura et al., 2021 [47]	2021	2019	International Journal of Clinical Pharmacy	Uganda	Eastern Africa	Cross-sectional	Non-receipt of prescribed antibiotics	Inpatient	0 and 59 months	427	Antibiotic prescribing
Skosana et al., 2022 [48]	2022	2018	Journal of Global Antimicrobial Resistance	South Africa	Southern Africa	Cross-sectional	Patients seen in emergency, day-case surgery, minor procedures, or chemotherapy	Inpatient	< 18 years	1261	Antibiotic prescribing AWaRe group
Ndayizeye et al., 2019 [49]	2019	2016	Rwanda Medical Journal	Rwanda	Eastern Africa	Cross-sectional	Neonates admitted from other sources (hospitals)	Inpatient	< 28 days of life	178	Antibiotic prescribing
Gres et al., 2024 [50]	2024	2021	BMJ Paediatrics Open	Burkina Faso, Guinea, Mali and Niger	Western Africa	Cross-sectional	Did not specify	Outpatient and Inpatient	< 5	346; 77; 285; e 260	Antibiotic prescribing
Xavier et al., 2024 [51]	2024	2019	*Scientific Reports*	Mozambique	Eastern Africa	Cross-sectional	Patients with missing essential information; microbiological indications not documented	Inpatient	≤ 10 years	307	Antibiotic prescribing AWaRe group
Gidey et al., 2024 [52]	2024	2021	Infection and Drug Resistance	Ethiopia	Eastern Africa	Cohort	Patients/caregivers who refused participation and patients readmitted during the study	Inpatient	≤ 15 years	232	Antibiotic prescribing
Mambula et al., 2023 [53]	2023	2019	Antimicrobial Resistance & Infection Control	Uganda and Niger	Eastern Africa and Western Africa	Cross-sectional	Did not specify	Outpatient and Inpatient	< 17	1622; 660	Antibiotic prescribing
Tusubira et al., 2023 [54]	2023	2017	African Journal of Primary Health Care & Family Medicine	Uganda	Eastern Africa	Cross-sectional	Children with conditions requiring antibiotics per Clinical Guidelines and those with caregivers < 18 years old	Outpatient	< 5	382	Antibiotic prescribing
Xavier et al., 2022 [55]	2022	2019	Antimicrobial Resistance & Infection Control	Mozambique	Eastern Africa	Cross-sectional	Patients with missing essential clinical information (age, sex, weight, diagnosis, outcome, dosage, route, duration, hospitalization)	Inpatient	≤ 10 years	315	Antibiotic prescribing
Allwell-Brown et al., 2022 [56]	2022	2021	JAC-Antimicrobial Resistance	Uganda	Eastern Africa	Cross-sectional	Records lacking documented fever or with incomplete/illegible diagnosis and prescription entries	Outpatient	< 5 years	3598	Antibiotic prescribing AWaRe classification
Garedow and Tesfaye, 2022 [57]	*2022*	2021	*Infection and drug resistance*	Ethiopia	Eastern Africa	Cross-sectional	Patients prescribed topical antimicrobials, antifungals, antivirals, anthelmintics, antiprotozoals, or antituberculosis drugs	Inpatient	≤ 18 years	6100	Antibiotic prescribing
Alekaw et al., 2022 [58]	2022	2021	Infection and Drug Resistance	Ethiopia	Eastern Africa	Cross-sectional	Patients who received topical/non-systemic antibiotics and received only anti-TB medications	Inpatient	≤ 14 years	279	Antibiotic prescribing
Kakolwa et al., 2021 [59]	2021	2018	*Antimicrobial Resistance & Infection Control*	Tanzania	Eastern Africa	Cross-sectional	Did not specify	Inpatient	neonates	162	Antibiotic prescribing
Sié et al., 2021 [60]	2021	2020	Clinical Infectious Diseases	Burkina Faso	Western Africa	Cross-sectional	Follow-up visits	Outpatient	< 5 years	**61,355**	Antibiotic prescribing
Yehualaw et al., 2021 [61]	2021	2019	*BioMed Research International*	Ethiopia	Eastern Africa	Cross-sectional	Patients using antibiotics for prophylaxis	Inpatient	< 18 years	692	Antibiotic prescribing
Garcia-Vello et al., 2020 [62]	2020	2015	Pan African Medical Journal	Ghana	Western Africa	Cross-sectional	Did not specify	Inpatient	Not reported	617	Antibiotic prescribing
Sié et al., 2019 [63]	2019	2017	American Journal of Tropical Medicine and Hygiene	Burkina Faso	Western Africa	Cross-sectional	Did not specify	Outpatient	< 5 years	1444	Antibiotic prescribing
Labi et al., 2018 [64]	2018	2016	BMC Pediatrics	Ghana	Western Africa	Cross-sectional	None	Inpatient	< 18 years	849	Antibiotic prescribing
Morgan et al., 2018 [65]	2018	2013	S. Afr. j. child health	South Africa	Southern Africa	Cross-sectional	Did not specify	Inpatient	Not reported	131	Antibiotic prescribing
Adisa et al., 2018 [66]	2018	2015	African Health Sciences	Nigeria	Western Africa	Cross-sectional	Mothers of children > 5 years and those who declined participation	Outpatient	< 5 years	243	Antibiotic prescribing
Umar et al., 2018 [67]	2018	2014	Annals of African Medicine	Nigeria	Western Africa	Cross-sectional	Prescriptions without client identity, repeated prescriptions, or revised prescriptions	Inpatient	< 18 years	3924	Antibiotic prescribing
Kebede et al., 2017 [68]	2017	2014	PLoS ONE	Ethiopia	Eastern Africa	Cross-sectional	Incomplete records	Inpatient	≤ 14 years	407	Antibiotic prescribing
Monteiro et al., 2017 [69]	2017	2015	South African Journal of Child Health (SAJCH)	Mozambique	Eastern Africa	Cross-sectional	Did not specify	Inpatient	0–14 years	454	Antibiotic prescribing
Koura et al., 2013 [70]	2013	2008	Acta Tropica	Benin	Western Africa	Cross-sectional	Did not specify	Outpatient	Not reported	1630	Antibiotic prescribing
Mabiala Babela et al., 2013 [71]	2013	2009	Médecine et Santé Tropicales	Republic of the Congo	Central Africa	Cross-sectional	Did not specify	Inpatient	≤ 16 years	464	Antibiotic prescribing
Gwimile et al., 2012 [72]	2012	2010	Pan African Medical Journal	Tanzania	Eastern Africa	Cross-sectional	Did not specify	Outpatient	< 5 years	384	Antibiotic prescribing
Pillay et al., 2024 [73]	2024	2021	Journal of Tropical Pediatrics	South Africa	Southern Africa	Cross-sectional	Patients previously recruited into the study, those receiving topical or ophthalmologic antibiotics, and those for discharge on the survey day	Inpatient	≤ 15 years	1191	Antibiotic prescribing
Rhee et al., 2019 [74]	2019	2012	BMC Public Health	Kenya	Eastern Africa	Cross-sectional	Records with non-diarrheal indications for antibiotics (per IMCI or discharge diagnoses), including serious infections and parasitic diseases (amoebiasis/giardiasis)	Outpatient	2-59 months	78,602	Antibiotic prescribing

^*^For studies with extended data collection periods spanning more than one year, a single approximate year was derived using the midpoint of the data collection period

**Table 2:** Subgroup analysis and meta-regression for sources of heterogeneity

Characteristics	No. of studies	Proportion (IC 95%)	*I*^2^ (%)	*p*-valor^a^	*p*-value^b^	Regression coefficient (95% CI); *p*-value	
						**Univariable**	**Multivariable**
**Total**	61	0.76 (0.68; 0.82)	99.8	*p* < 0.001		--------------------------	--------------------------
**Risk of bias**							
Fair	56	0.74 (0.66; 0.81)	99.8	*p* < 0.001	*p* = 0.136	Ref	
Good	3	0.85 (0.53; 0.99)	99.6	*p* < 0.001		0.14 (-0.227; 0.514); 0.447	0.17 (*-*0.170; 0.508); 0.323
Poor	2	0.99 (0.73; 1.00)	64.3	*p* < 0.001		0.43 (*-*0.019; 0.884); 0.061	0.56 (0.184; 0.931); 0.004
**Study design**							
Cross-sectional	56	0.76 (0.68; 0.83)	99.8	*p* < 0.001	*p* = 0.929	Ref	
Cohort	5	0.77 (0.50; 0.95)	99.5	*p* < 0.001		*-*0.01 (*-*0.308; 0.282); 0.929	--------------------------
**Size**							
< 200	26	0.84 (0.73; 0.92)	95.4	*p* < 0.001	*p* = 0.160	Ref	
200-500	14	0.72 (0.56; 0.86)	99.2	*p* < 0.001		*-*0.13 (*-*0.339; 0.087); 0.247	*-*0.08 (*-*0.257; 0.089); 0.335
> 500	21	0.68 (0.54; 0.80)	99.9	*p* < 0.001		*-*0.18 (*-*0.369; 0.009); 0.062	*−*0.15 (*−*0.303; *−*0.002); 0.046
**Type of patient**							
Both	1	0.85 (0.23; 1.00)	––	*p* < 0.001		Ref	
Inpatient	36	0.79 (0.69; 0.88)	99.7	*p* < 0.001	*p* = 0.500	*-*0.08 (*-*0.759; 0.600); 0.819	--------------------------
Outpatient	24	0.70 (0.57; 0.82)	99.9	*p* < 0.001		*-*0.18 (*-*0.865; 0.503); 0.603	--------------------------
**Geographic region**							
Eastern Africa	16	0.64 (0.49; 0.77)	99.8	*p* < 0.001	*p* = 0.083	Ref	
Western Africa	34	0.83 (0.74; 0.91)	99.8	*p* < 0.001		1.07 (0.4355; 1.704); < 0.001	0.23 (0.069; 0.391); 0.006
Central Africa	2	0.85 (0.44; 1.00)	99.8	*p* < 0.001		1.65 (0.059; 3.251); 0.042	0.27 (*-*0.124; 0.673); 0.173
Southern Africa	6	0.64 (0.38; 0.86)	95.1	*p* < 0.001		*-*0.05 (*-*1.053; 0.963); 0.929	0.01 (-0.233; 0.258); 0.919
**Study period** (median 2018 (IQR 2016-2021), range 2006-2022)							
≤ 2014	12	0.60 (0.41; 0.78)	99.6	*p* < 0.001	p = 0.081	0.024 (0.002; 0.046); 0.0311	0.01 (*-*0.004; 0.033); 0.126
2015-2019	26	0.74 (0.61; 0.85)	99.3	*p* < 0.001		--------------------------	---------------------------
≥ 2020	23	0.85 (0.73; 0.94)	99.9	*p* < 0.001		--------------------------	---------------------------

Legend: ^a^Within groups; ^b^Test for subgroup differences (Between)

### Meta-analysis

#### Patterns of antibiotic prescription

**Fig. 3:**
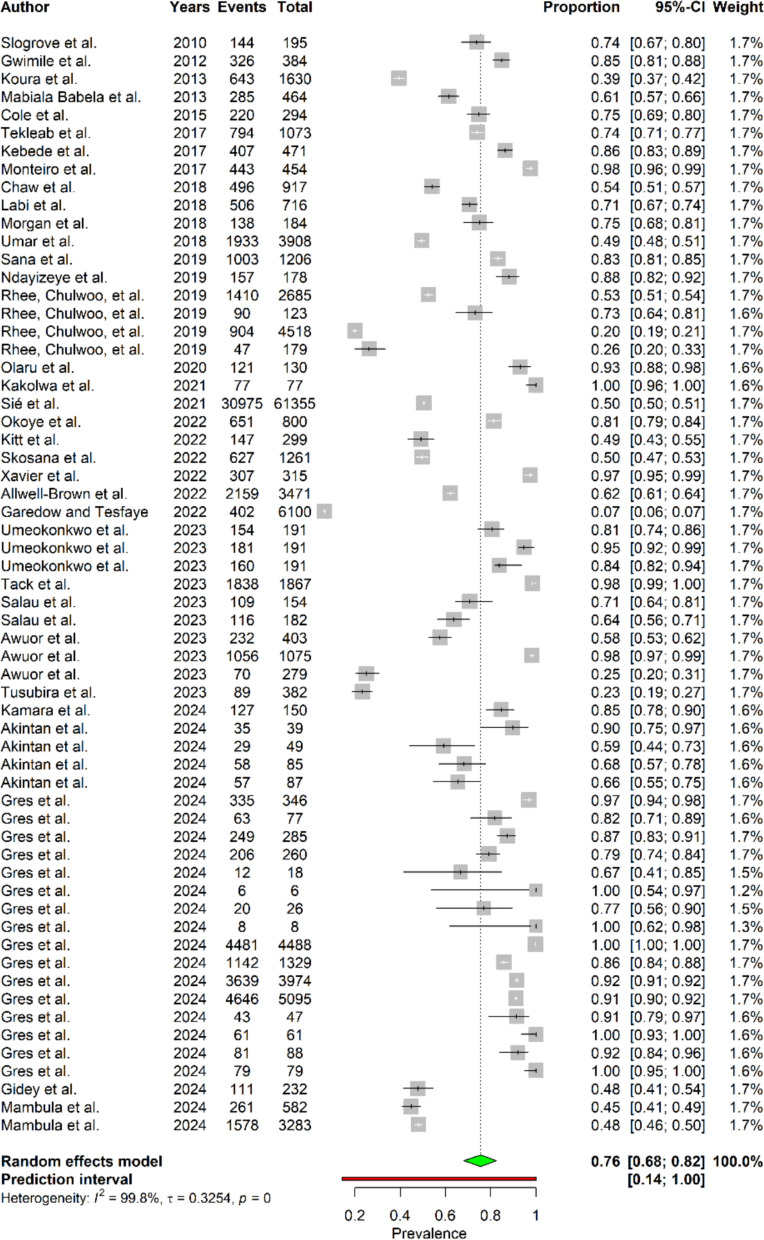
Forest plot of pooled patient-level prevalence of antibiotic prescribing in children in Africa

As shown in Fig. 3, a total of 61 studies reported patient-level prevalence of antibiotic prescription. Using the Freeman-Tukey double arcsine transformation, the pooled prevalence was 76.0% (95% CI, 68.1-82.4). Extreme heterogeneity was observed across studies (*I*^2^ = 99.8%; *p* < 0.001).

#### Routes of administration and mean number of antibiotics

**Fig. 4:**
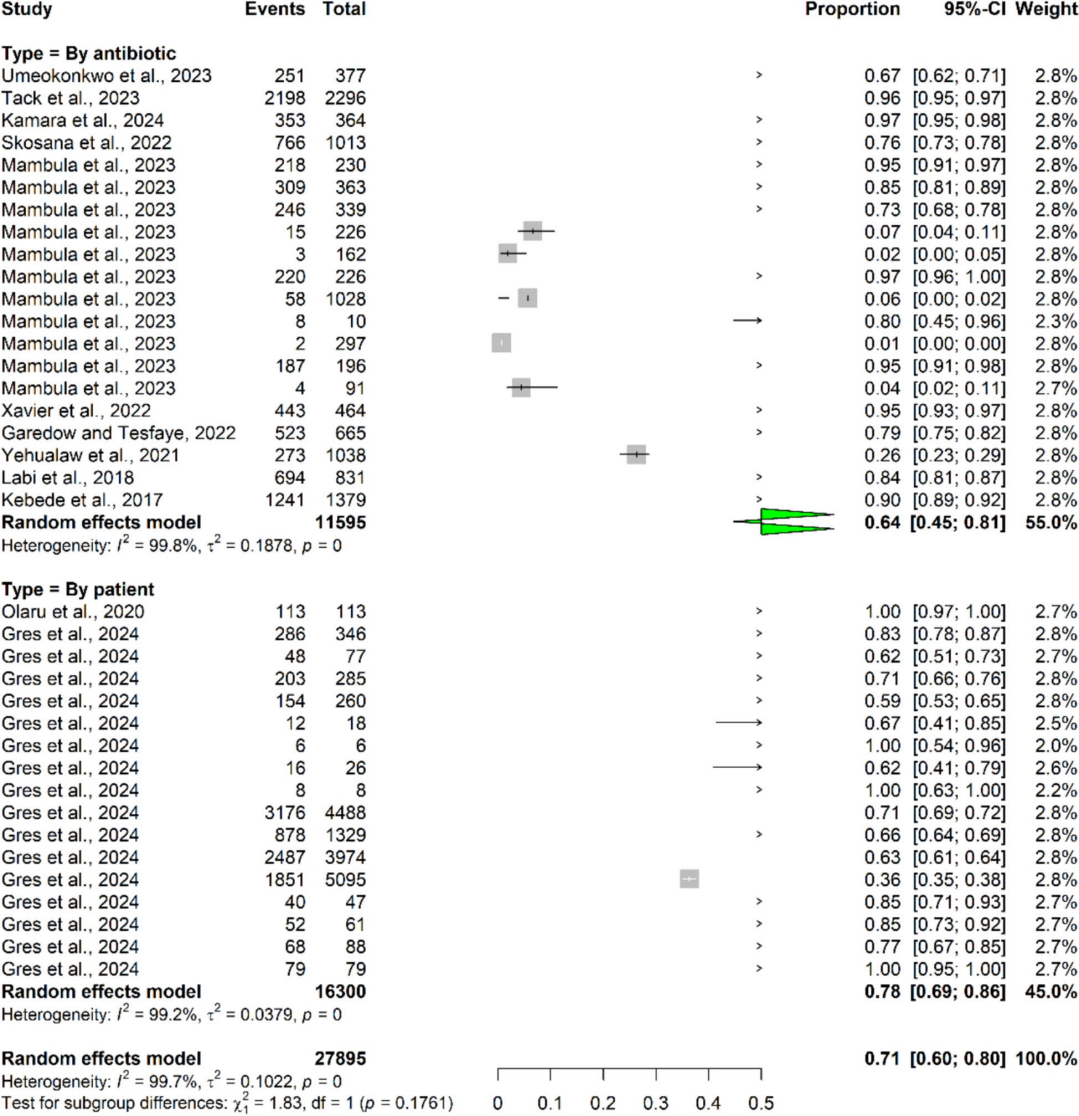
Forest plot of parenteral antibiotic use at the patient and prescription levels

**Fig. 5:**
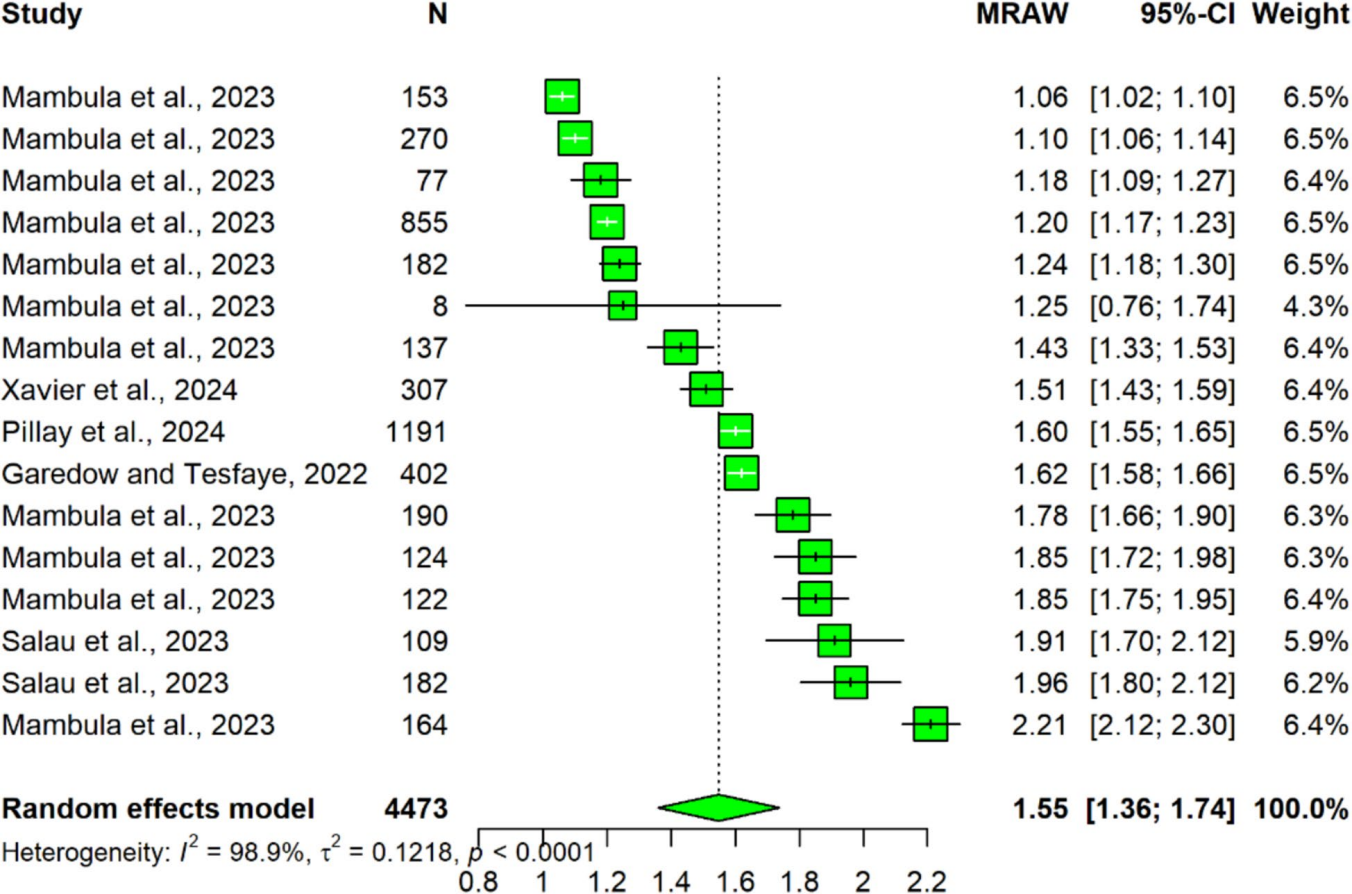
Pooled mean number of antibiotic prescriptions per patient

Most antibiotic use involved parenteral administration. At the patient level, 78.0% of children received at least one parenteral antibiotic, whereas at the prescription level, parenteral formulations accounted for 64.0% of all antibiotics prescribed (Fig. 4). The mean number of antibiotics prescribed per patient was 1.55 (95% CI, 1.36-1.74; *I*^2^ = 98.9%), with estimates ranging from 1.06 to 2.21 across studies (Fig. 5).

### Subgroup and meta-regression analysis

**Fig. 6:**
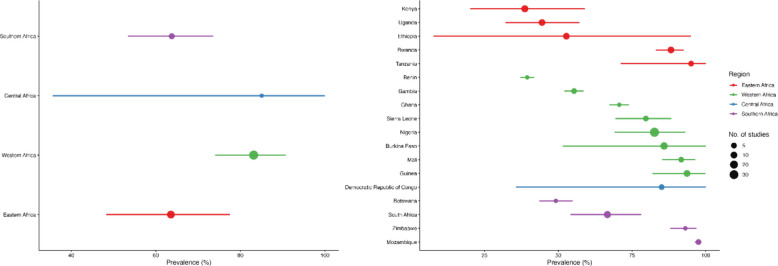
Distribution of antibiotic prescription prevalence across regions and countries

No statistically significant differences were observed by study design, with pooled prevalence estimates of 77.0% in cohort studies and 76.0% in cross-sectional studies (*p* = 0.929). Regarding sample size, studies including fewer than 200 participants reported higher pooled prevalence estimates (84.0%) compared with studies enrolling 200-500 participants (72.0%) and more than 500 participants (68.0%), although subgroup differences were not statistically significant (*p* = 0.160) (Fig. S2. Fig. 1-6).

Similarly, pooled prevalence estimates did not differ significantly between inpatient and outpatient settings (79.0% vs. 70.0%, respectively; *p* = 0.500). Across regions, prevalence estimates were higher in Central Africa (85.0%) and Western Africa (83.0%) compared with Eastern and Southern Africa (64.0%), but subgroup differences did not reach statistical significance (*p* = 0.083). With respect to study period, pooled prevalence estimates increased from 60.0% in studies with data collected up to 2014 to 74.0% in 2015-2019 and 85.0% from 2020 onwards. Nevertheless, subgroup differences by study period were not statistically significant (*p* = 0.081) (Fig. 6; S2. Figure 1-6).

In univariable meta-regression analyses, risk of bias and geographic region were identified as potential sources of heterogeneity. Compared with studies of fair quality, studies with poor methodological quality showed a higher pooled prevalence (coefficient, 0.43; *p* = 0.061). Geographic region was significantly associated with prevalence in the univariable model, with studies conducted in Western Africa showing higher prevalence compared with Eastern Africa (coefficient = 1.07; *p* < 0.001); studies from Central Africa also demonstrated a significant association (coefficient = 1.65; *p* = 0.042). In addition, study period showed a modest but statistically significant increasing trend in prevalence over time (coefficient = 0.024; *p* = 0.031) (Table 2).

In the multivariable meta-regression model, after adjustment for potential confounders, poor methodological quality remained independently associated with higher prevalence (coefficient, 0.56; *p* = 0.004). Likewise, studies conducted in Western Africa continued to show significantly higher prevalence compared with Eastern Africa (coefficient, 0.23; *p* = 0.006), whereas associations for Central Africa and study period were attenuated and no longer statistically significant (Table 2).

### Publication bias and sensitivity analysis

**Fig. 7:**
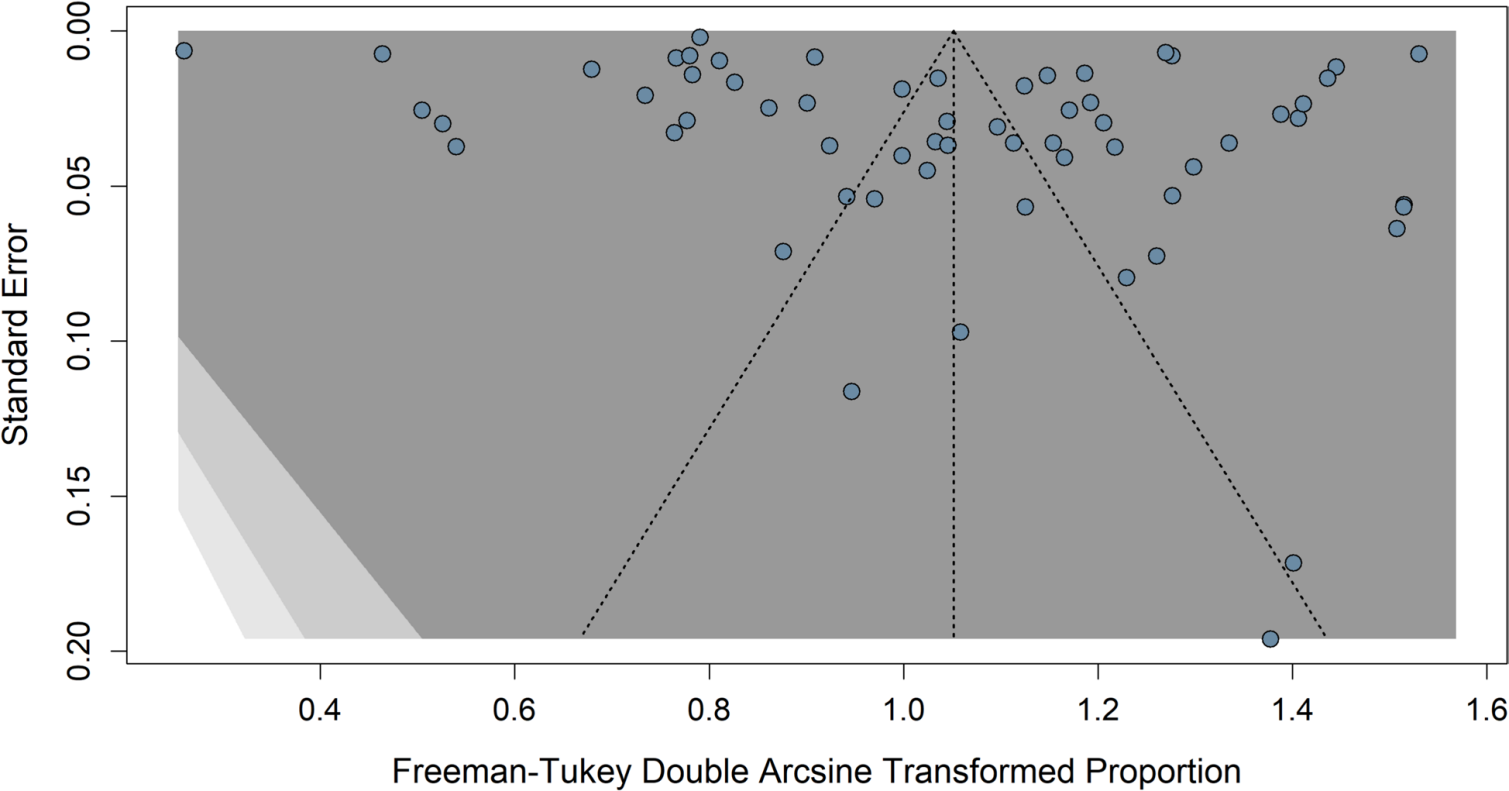
Funnel chart for evaluating publication channels

**Fig. 8:**
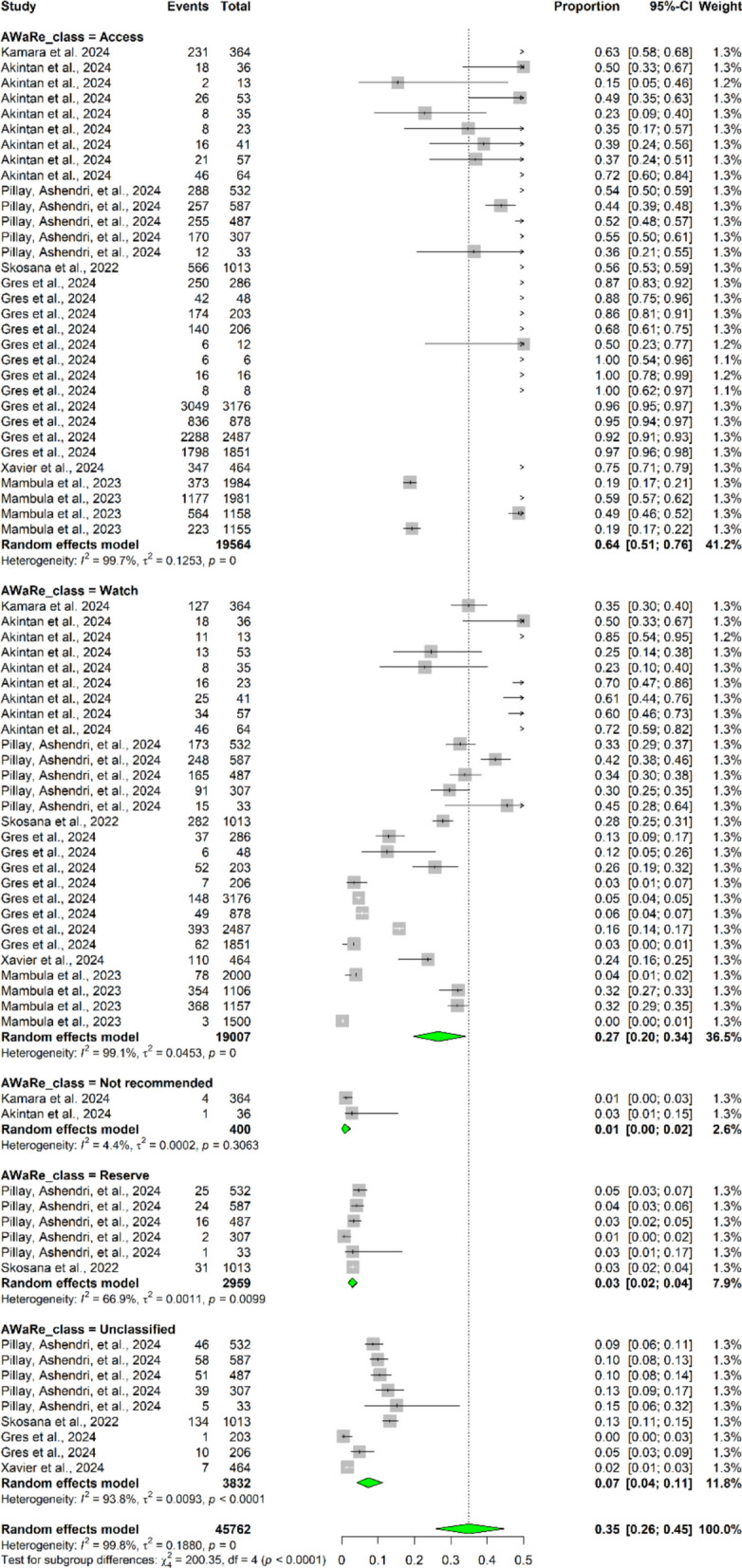
Antibiotic prescription pattern by AWaRe group

Publication bias was evaluated using funnel plots and Egger’s and Begg’s tests. Begg’s test was not statistically significant (*z* = -0.45; *p* = 0.654), whereas Egger’s test indicated significant asymmetry (*t* = 2.39; *p* = 0.020) (Fig. 7). Using the trim-and-fill method, 25 studies were imputed, resulting in an adjusted pooled prevalence estimate of 0.53 (95% CI, 0.47-0.60) (S2. Figure 7). When applying a random-effects trim-and-fill procedure with the Freeman-Tukey double arcsine transformation, 26 studies were imputed, yielding an adjusted pooled prevalence estimate of 0.51 (95% CI, 0.42-0.60). The leave-one-out sensitivity analysis indicated that no single study exerted a disproportionate influence on the pooled estimate (S2. Figure 8).

### WHO AWaRe classification

#### Access category

**Fig. 9:**
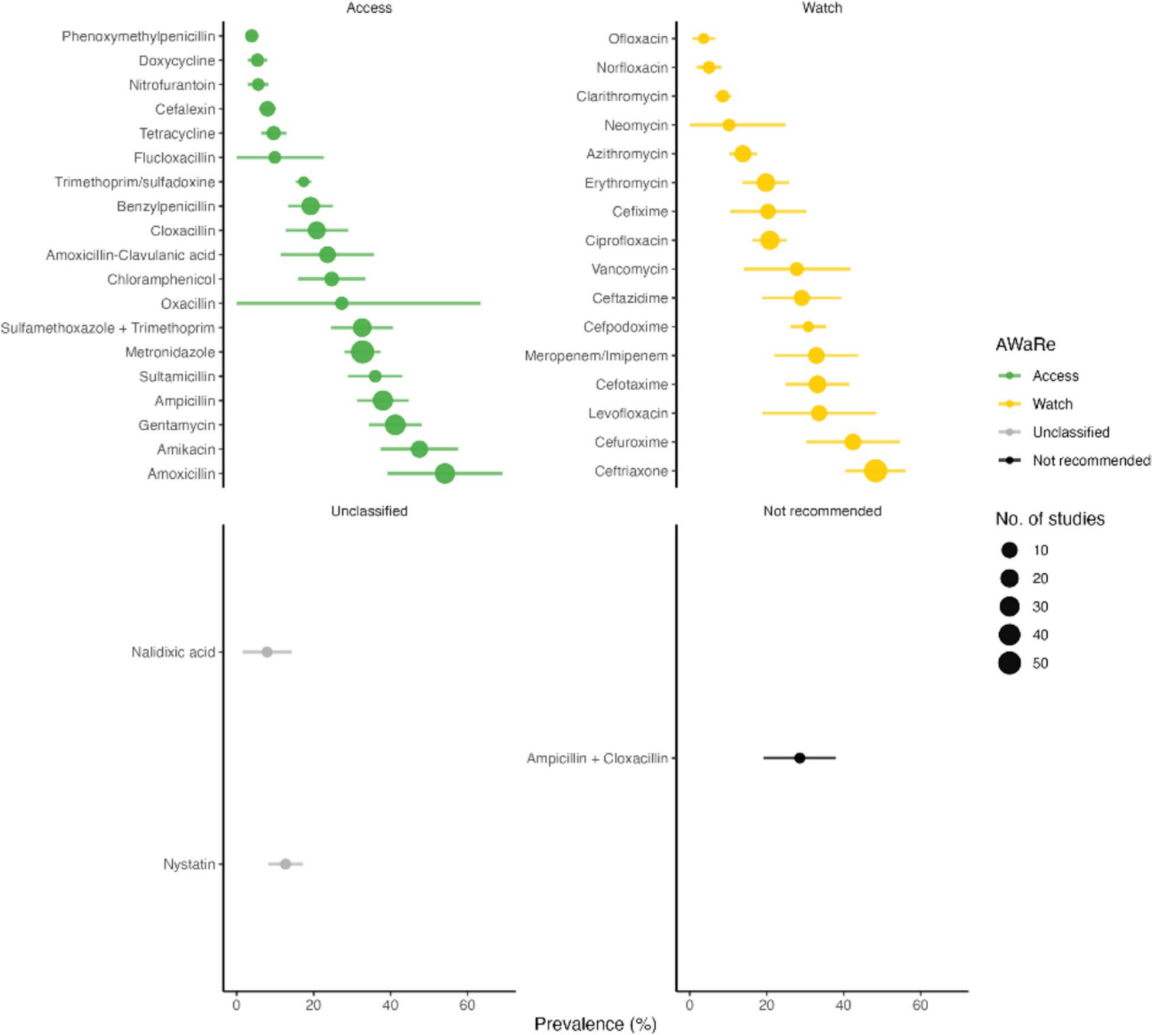
Distribution of antibiotics by AWaRe group-Freeman-Tukey

Analysis based on the WHO AWaRe classification showed that Access antibiotics accounted for a pooled prevalence of 65.0% (95% CI, 53.0; 75.0; *I*^2^ = 99.7%) (Fig. 8). The highest regional rates were observed in Western Africa (0.71; 95% CI, 0.54; 0.84), followed by Southern Africa (0.51; 95% CI, 0.47; 0.56) and Eastern Africa (0.50; 95% CI, 0.19; 0.81). At the country level, prevalence was highest in Guinea (0.95; 95% CI, 0.88; 1.00), Burkina Faso (0.87; 95% CI, 0.73; 0.97), Mali (0.92; 95% CI, 0.85; 0.97), and Mozambique (0.75; 95% CI, 0.71; 0.79), while the lowest value was reported in Niger (0.33; 95% CI, 0.09; 0.64). Moreover, Access antibiotics were more frequently prescribed in outpatients (0.82; 95% CI, 0.64; 0.94) than in inpatients (0.53; 95% CI, 0.44; 0.62; *p* = 0.005) (S2. Figure 9-11).

#### Watch category

The Watch group accounted for a pooled prevalence of 27.0% (95% CI, 20.0; 34.0; *I*^2^ = 99.1%) (Fig. 8). At the regional level, the highest prevalence was reported in Southern Africa (0.34; 95% CI, 0.29; 0.39), followed by Western Africa (0.25; 95% CI, 0.17; 0.34) and Eastern Africa (0.18; 95% CI, 0.02; 0.43). At the country level, the highest rates were observed in Mali (0.20; 95% CI, 0.11; 0.31), Mozambique (0.24; 95% CI, 0.20; 0.28), South Africa (0.34; 95% CI, 0.29; 0.39), and Nigeria (0.41; 95% CI, 0.20; 0.65), whereas Uganda (0.15; 95% CI, 0.00; 0.51), Guinea (0.08; 95% CI, 0.02; 0.16), and Burkina Faso (0.08; 95% CI, 0.02; 0.18) reported the lowest values. Prevalence was significantly higher among inpatients (0.39; 95% CI, 0.29; 0.50) compared with outpatients (0.09; 95% CI, 0.04; 0.17; *p* < 0.0001) (S2. Figure 12–14).

#### Reserve, unclassified and not recommended categories

Reserve antibiotics were reported in only one study, conducted in South Africa among inpatients, with a prevalence of 3.0% (95% CI, 2.0;–4.0%) (Fig. 8 and S2. Figure 1517). Unclassified antibiotics had a pooled prevalence of 7.0% (95% CI, 4.0-11.00%; *I*^2^ = 93.8%), with the highest rates in Southern Africa (0.11; 95% CI, 0.09-0.13), which is South Africa. Prevalence was higher among inpatients (0.09; 95% CI, 0.08-0.13) compared with outpatients (0.02; 95% CI, 0.02-0.0, *p* = 0.048) (Fig. 8 and S2. Figure 18–20). Not Recommended antibiotics were identified in two studies from Western Africa, with a pooled prevalence of 1.0% (95% CI, 0.00-2.00%; *I*^2^ = 4.4%), showing similar values in Sierra Leone and Nigeria (*p* = 0.306). All data in this category came from inpatients (Fig. 8 and S2. Figure 21–23).

AWaRe classification for Central Africa could not be assessed because the included studies from this region did not report sufficient information to allow classification of prescribed antibiotics.

#### Prevalence of specific antibiotics within AWaRe categories

Across specific agents, the most frequently prescribed Access antibiotics were amoxicillin (54.1%, 95% CI, 39.2; 69.0), amikacin (47.5%, 95% CI, 37.4; 57.5), gentamicin (41.2%, 95% CI, 34.4; 48.0), and ampicillin (38.0%, 95% CI, 31.3-44.7). In the Watch category, ceftriaxone (48.3%, 95% CI, 40.6-56.1), cefuroxime (42.4%, 95% CI, 30.2–54.6), and imipenem/meropenem (32.9%, 95% CI, 22.0; 43.8) predominated. In the not recommended category, ampicillin plus cloxacillin was reported at 28% (95% CI, 19.2-38.0) (Fig. 9; S2. Table 1).

## Discussion

This systematic review and meta-analysis synthesizes evidence on antibiotic prescribing patterns among pediatric patients in Africa. The pooled patient-level prevalence estimate was 76%, although heterogeneity across studies was extreme (*I*^2^ = 99.8%). Such heterogeneity indicates substantial variability in study populations, healthcare settings, prescribing practices, and measurement approaches. Therefore, the pooled estimate should be interpreted cautiously as a descriptive aggregate rather than a precise estimate of underlying prevalence.

Regional analyses suggested variability in prescribing prevalence, with higher estimates reported in Central (85.0%) and Western Africa (83.0%). However, subgroup differences were not statistically significant, and these findings should be interpreted with caution given the high between-study heterogeneity.

Subgroup analyses and meta-regression indicated that methodological quality, geographic region, sample size, and year of publication contributed to between-study variability. These findings suggest that contextual, methodological, and temporal factors may influence the reported prevalence of antibiotic prescribing.

When compared with global evidence, our pooled prevalence estimate is broadly consistent with a multicenter study conducted across 59 low- and middle-income countries, which reported a prevalence of 74% [75]. In contrast, substantially lower prevalence rates have been consistently reported in high-income settings. In Europe, antibiotic use among pediatric patients has been reported at approximately 32% [76, 77].

A multicenter study comparing European and non-European hospitals further showed significantly higher antibiotic use in non-European pediatric (43.8%) and neonatal (39.4%) populations compared with their European counterparts (35.4% and 21.8%, respectively) [78]. Similarly, lower prescription rates have been documented in other high-income countries, including the USA (25.9%) [79], Japan (7.4%) [80], and China (27.1%) [81]. Within Asia, substantial heterogeneity in antibiotic use has also been observed, with prescription rates ranging from 65.75% in Northern India to 36.77% in Eastern India [82, 83]. Nevertheless, cross-study comparisons should be interpreted cautiously due to differences in study design, populations, case mix, and measurement methods.

Although variability across regions and settings was considerable, antibiotic prescribing prevalence in African pediatric populations appears relatively high compared with estimates reported in many high-income countries. However, the extreme heterogeneity observed limits firm conclusions regarding the magnitude of these differences.

When interpreted in relation to WHO reference values, the pooled prevalence estimate of antibiotic prescribing and the proportion of children receiving at least one parenteral antibiotic (78.0%) appear higher than the ranges reported in WHO benchmarking studies (2-26.8% for prescribing prevalence and 13.4-24.1% for parenteral use) [84]. Nevertheless, these comparisons should be interpreted cautiously, as WHO benchmarks are context-specific and differences in study design, patient populations, clinical severity, and healthcare infrastructure may affect comparability.

This pattern of antibiotic prescribing in pediatric populations may have implications for antibiotic effectiveness and AMR [75]. AMR is widely recognized as one of the most serious threats to human health [1], and inappropriate or unnecessary antibiotic use is considered an important contributing factor [7]. However, prescribing practices are influenced by multiple interacting determinants.

Factors that may contribute to potentially inappropriate prescribing include diagnostic uncertainty, perceived clinical risk, expectations from caregivers, and variability in local prescribing practices [85]. Structural limitations within healthcare systems, such as restricted diagnostic capacity, limited resources, and constrained access to alternative therapeutic options, may also shape antibiotic use [86, 87]. The fragility of health systems, reflected in the type of institution and the absence of formal national guidelines, may further contribute to variability in prescribing patterns [88]. Additionally, the available evidence shows geographic gaps, with uneven representation across sub-Saharan Africa. This uneven coverage may influence regional estimates and limits the generalizability of the pooled findings.

With respect to the AWaRe classification, the highest frequency of prescriptions was observed in the Access group (65.0%), particularly in Western Africa (71.0%) and among outpatients. The Watch group accounted for 27.0% of prescriptions, being more common in Southern Africa (34.0%) and mainly among inpatients (39.0%). The Reserve group represented 3.0% of prescriptions, all reported in South Africa and exclusively among inpatients.

These findings are broadly consistent with a study conducted in 56 countries including children under 19 years, in which Africa had the highest proportion of Access antibiotics (around 69% in children and 40% in neonates). In that study, neonates received proportionally more Watch antibiotics than older children [3].

In the present study, the most frequently prescribed antibiotics were amoxicillin, amikacin, and gentamicin in the Access group; ceftriaxone and cefuroxime in the Watch group; and ampicillin + cloxacillin in the Not recommended group.

These patterns align with the global literature, which highlights ceftriaxone as one of the most commonly prescribed antibiotics worldwide, not only in Africa but also in regions such as the Eastern Mediterranean, Europe, and Southeast Asia [3]. The relatively low use of Reserve and Not Recommended antibiotics may reflect partial implementation of stewardship principles, though caution is warranted in interpreting this as direct evidence of adherence. Similarly, the observed mean number of antibiotics per patient highlights potential concerns regarding polypharmacy and its implications for antimicrobial resistance. These findings should be interpreted within the context of local healthcare practices, national policies, and ongoing efforts to harmonize protocols and strengthen surveillance of antibiotic use.

The AWaRe classification is an important tool for monitoring and optimizing antibiotic use [89]. According to WHO guidance, at least 60% of prescriptions should belong to the Access group [90].

In this review, Access antibiotics accounted for the largest share of prescriptions overall, with regional variability. In some regions, the proportion of Access antibiotics exceeded this benchmark. This distribution may reflect the high burden of common childhood infectious syndromes, including pneumonia, persistent cough, diarrheal diseases, malaria, and fever [14], which are frequently managed empirically [91]. Some studies suggest that a substantial proportion of prescribed antibiotics, possibly more than 40%, may have been unnecessary [2]. Although WHO does not define specific thresholds for Watch antibiotics, our results suggest relatively high use, particularly in Southern Africa and among inpatients. These observations highlight the potential value of antimicrobial stewardship programs and the reinforcement of adherence to existing guidelines as strategies to support rational antibiotic use [92].

High levels of antibiotic prescribing in pediatric populations may have implications for morbidity, mortality, and antimicrobial resistance, as well as financial burdens on health systems, particularly in sub-Saharan Africa [93, 94]. Projections indicate that, if current trends persist, AMR could lead to substantial global health impacts in the coming decades [95].

Contributing factors include limited access to laboratories capable of performing cultures and antimicrobial susceptibility testing, gaps in regulatory oversight, and lack of systematic monitoring of antibiotic use. These conditions may encourage empiric prescribing and repeated use of broad-spectrum antibiotics, potentially increasing the risks associated with AMR [87].

This review has several limitations that should be considered. First, substantial heterogeneity was observed across the included studies, partly explained by differences in methodological quality, geographic region, and year of publication. These findings suggest that pooled prevalence estimates should be interpreted with caution, serving primarily as descriptive indicators of overall prescribing patterns rather than precise measures of underlying prevalence.

Publication bias represents another potential limitation. Funnel plots and Egger’s and Begg’s tests suggested possible asymmetry. Using the trim-and-fill method, 25-26 studies were imputed, resulting in adjusted pooled prevalence estimates that were substantially lower (0.51-0.53) than the original values. This notable shift underscores the instability of pooled prevalence estimates and highlights that the findings may be influenced by both heterogeneity and selective reporting.

Second, the small sample size in some studies may have introduced bias, potentially leading to overestimation of pooled estimates. In addition, publication bias represents a possible limitation; although statistical techniques were applied to mitigate it, the original results were retained in the analyses. Another important limitation relates to data availability and reporting quality.

Several potentially relevant variables, including clinical indications, patient age strata, neonatal status, and the presence of antimicrobial stewardship programs, were frequently missing, inconsistently reported, or not comparable across studies, which precluded their inclusion in subgroup analyses or multivariable meta-regression. Geographic representativeness also remains limited, as some countries and regions in Africa lack published studies or sufficiently detailed reporting. This may affect the generalizability of the findings across the continent and restrict regional comparisons, particularly for AWaRe classification in Central Africa.

Despite these limitations, this review provides valuable descriptive evidence on antibiotic prescribing patterns in pediatric populations in Africa, including the distribution according to AWaRe classification and the main antibiotics prescribed. Future studies should expand geographic coverage, assess prescription appropriateness relative to clinical guidelines, explore social and institutional determinants of antibiotic use, and evaluate the impact of stewardship interventions in African pediatric settings. Considering the high heterogeneity and potential publication bias, such evidence may better inform context-specific health policies and support the rational use of antibiotics.

## Conclusion

This systematic review and meta-analysis provides descriptive evidence of high antibiotic prescription prevalence in children across Africa, with notable regional variation and particularly elevated rates in Central, Western, and Eastern Africa. While Access antibiotics accounted for the majority of prescriptions, substantial use of Watch antibiotics was observed, especially among inpatients. These findings likely reflect a combination of high pediatric infectious disease burden, diagnostic constraints, and structural limitations within healthcare systems. Given the observed heterogeneity and potential publication bias, the pooled prevalence estimates should be interpreted with caution. The evidence underscores the potential value of strengthening antimicrobial stewardship programs tailored to the African context. Key strategies may include supporting rational use of Access antibiotics, limiting unnecessary use of Watch antibiotics, enhancing laboratory capacity, implementing national guidelines, and monitoring antibiotic use systematically to align clinical practice with WHO recommendations.

## Supplementary Information

Supplementary Material 1: S1. Search strategy.

Supplementary Material 2: S2. Antibiotic use, ATC codes, and AWaRe classification with pooled prevalence and heterogeneity; subgroup analyses; funnel plot using the trim-and-fill method; sensitivity analysis of the prevalence of antibiotic prescriptions; forest plot of AWaRe groups; and NIH Quality Assessment Tool for Observational Cohort and Cross-Sectional Studies.

Supplementary Material 3: S3. DerSimonian–Laird random-effects.

Supplementary Material 4: S4. PRISMA Checklist.

## Data Availability

The datasets used and/or analyzed during the current study are available from the corresponding author upon reasonable request.
