# Supplementary material for: National and regional challenges of antibiotic prescribing patterns in pediatric populations across Africa: a systematic review and meta-analysis
Source: Syst Rev. 2026 Jun 10;15:228. doi: 10.1186/s13643-026-03223-9 (PMC13480107; doi:10.1186/s13643-026-03223-9)
Supplement: Supplementary file 1 [file 13643_2026_3223_MOESM1_ESM.docx]

**Supplementary material (S1)**

**S1. Table 1.** Search strategy

| Nr. | **Databases** | **Syntex** | **Articles** |
| --- | --- | --- | --- |
| 1 | Pubmed | (("pediatrics"[MeSH Terms] OR "child"[MeSH Terms] OR "infant, newborn"[MeSH Terms] OR "infant"[MeSH Terms]) AND ((("anti-bacterial agents"[All Fields] OR "anti-bacterial agents"[Supplementary Concept] OR "anti-bacterial agents"[All Fields] OR "antibiotic"[All Fields] OR "anti-bacterial agents"[MeSH Terms] OR ("anti-bacterial"[All Fields] AND "agents"[All Fields])) AND prescribing[All Fields] AND ("Patterns (N Y)"[Journal] OR "patterns"[All Fields])) OR ("anti-bacterial agents"[All Fields] OR "anti-bacterial agents"[Supplementary Concept] OR "anti-bacterial agents"[All Fields] OR "antibiotic"[All Fields] OR "anti-bacterial agents"[MeSH Terms] OR ("anti-bacterial"[All Fields] AND "agents"[All Fields])) OR (("anti-bacterial agents"[All Fields] OR "anti-bacterial agents"[Supplementary Concept] OR "anti-bacterial agents"[All Fields] OR "antibiotic"[All Fields] OR "anti-bacterial agents"[MeSH Terms] OR ("anti-bacterial"[All Fields] AND "agents"[All Fields])) AND ("prescriptions"[MeSH Terms] OR "prescriptions"[All Fields] OR "prescription"[All Fields])) OR ("anti-bacterial agents"[All Fields] OR "anti-bacterial agents"[Supplementary Concept] OR "anti-bacterial agents"[All Fields] OR "antibiotic"[All Fields] OR "anti-bacterial agents"[MeSH Terms] OR ("anti-bacterial"[All Fields] AND "agents"[All Fields])))) AND ("africa"[MeSH Terms] OR "africa"[All Fields]) | 8711 |
| 2 | Embase | ('pediatric'/exp OR 'pediatric' OR 'children'/exp OR 'children' OR 'neonates' OR 'infants'/exp OR 'infants') AND ('antibiotic prescribing patterns' OR 'antibiotic use'/exp OR 'antibiotic use' OR 'antibiotic prescription'/exp OR 'antibiotic prescription' OR 'use of antibiotic') AND ('africa'/exp OR 'africa') | 590 |
| 3 | Scopus | TITLE-ABS-KEY((Pediatric OR children OR neonates OR infants) AND (Antibiotic prescribing patterns OR Antibiotic use OR antibiotic prescription OR Antimicrobial resistance) AND (Africa OR Sub-Saharan Africa OR North Africa)) | 225 |
| 4 | Web of Science | TS=("Pediatric" OR "children" OR "neonates" OR "infants") AND TS=("Antibiotic prescribing patterns" OR "Antibiotic use" OR "Antibiotic prescription" OR "Antimicrobial resistance") AND TS=("Africa" OR "Sub-Saharan Africa" OR "North Africa") | 200 |
| 5 | WHO Global Index Medicus | ( "Pediatric" OR "children" OR "neonates" OR "infants" ) AND ( "Antibiotic prescribing patterns" OR "Antibiotic use" OR "Antibiotic prescription" OR " use of antibiotic" ) AND ( "Africa" ) | 9 |
| 6 | Geral* | ( "Pediatric" OR "Paediatric" OR "children" OR "neonates" OR "infants" ) AND ( "Antibiotic prescribing patterns" OR "Antibiotic use" OR "Antibiotic prescription" OR " use of antibiotic" ) AND ( "Africa" ) |  |

*Search in databases such as WHO Global Index Medicus
