# Supplementary material for: National and regional challenges of antibiotic prescribing patterns in pediatric populations across Africa: a systematic review and meta-analysis
Source: Syst Rev. 2026 Jun 10;15:228. doi: 10.1186/s13643-026-03223-9 (PMC13480107; doi:10.1186/s13643-026-03223-9)
Supplement: Supplementary file 2 [file 13643_2026_3223_MOESM2_ESM.docx]

**Supplementary material (S2)**

**S2. Table 1.** Antibiotic use, ATC code, and AWaRe classification with pooled prevalence

| **Antibiotics** | **ATC Code** | **AWaRe Class** | **Pooled prevalence** | **95% CI** | **i2** | **No. of studies** |
| --- | --- | --- | --- | --- | --- | --- |
| Amoxicillin | J01CA04 | Access | 54.1 | 39.2; 69 | 0.999 | 32 |
| Ceftriaxone | J01DD04 | Watch | 48.3 | 40.6; 56.1 | 0.995 | 52 |
| Amikacin | J01GB06 | Access | 47.5 | 37.4; 57.5 | 0.893 | 15 |
| Cefuroxime | J01DC02 | Watch | 42.4 | 30.2; 54.6 | 0.985 | 12 |
| Gentamycin | J01GB03 | Access | 41.2 | 34.4; 48 | 0.992 | 34 |
| Ampicillin | J01CA01 | Access | 38 | 31.3; 44.7 | 0.992 | 31 |
| Sultamicillin | J01CR04 | Access | 36 | 28.9; 43.1 | 0.332 | 2 |
| Levofloxacin | J01MA12 | Watch | 33.6 | 18.8; 48.4 | 0.902 | 10 |
| Cefotaxime | J01DD01 | Watch | 33.2 | 24.8; 41.5 | 0.980 | 17 |
| Meropenem/Imiperem | J01DH02 | Watch | 32.9 | 22; 43.8 | 0.923 | 12 |
| Metronidazole | P01AB01 | Access | 32.7 | 28; 37.4 | 0.990 | 52 |
| Sulfamethoxazole + Trimethoprim | J01EE01 | Access | 32.6 | 24.5; 40.6 | 0.996 | 22 |
| Cefpodoxime | J01DD13 | Watch | 30.8 | 26.2; 35.4 | 0.000 | 1 |
| Ceftazidime | J01DD02 | Watch | 29.1 | 18.8; 39.4 | 0.955 | 10 |
| Ampicillin + Cloxacillin | J01CA51 | Not recommended | 28.6 | 19.2; 38 | 0.971 | 4 |
| Vancomycin | J01XA01 | Watch | 27.8 | 14; 41.7 | 0.962 | 4 |
| Oxacillin | J01CF02 | Access | 27.3 | 0.001; 63.4 | 0.9961 | 3 |
| Chloramphenicol | D06AX02 | Access | 24.7 | 16; 33.5 | 0.973 | 6 |
| Amoxicillin-Clavulanic acid | J01CR02 | Access | 23.6 | 11.5; 35.7 | 0.995 | 13 |
| Ciprofloxacin | J01MA02 | Watch | 20.8 | 16.3; 25.2 | 0.986 | 24 |
| Cloxacillin | J01CF02 | Access | 20.8 | 12.7; 29 | 0.991 | 18 |
| Cefixime | J01DD08 | Watch | 20.3 | 10.5; 30.2 | 0.988 | 8 |
| Erythromycin | J01FA01 | Watch | 19.8 | 13.7; 25.8 | 0.993 | 21 |
| Benzylpenicillin | J01CE01 | Access | 19.2 | 13.4; 25 | 0.989 | 20 |
| Trimethoprim/sulfadoxine | J01EE03 | Access | 17.4 | 15.4; 19.4 | 0.000 | 1 |
| Azithromycin | J01FA10 | Watch | 13.8 | 10.2; 17.5 | 0.944 | 14 |
| Nystatin | D01AA01 | Unclassified | 12.7 | 8.2; 17.3 | 0.000 | 1 |
| Neomycin | A07AA | Watch | 10.2 | 0.001; 24.9 | 0.985 | 2 |
| FluCloxacillin | J01CF05 | Access | 9.9 | 0.001; 22.5 | 0.985 | 2 |
| Tetracycline | J01AA07 | Access | 9.6 | 6.4; 12.9 | 0.822 | 5 |
| Clarithromycin | J01FA09 | Watch | 8.6 | 6.5; 10.8 | 0.000 | 2 |
| Cefalexin | J01DB01 | Access | 8 | 5.8; 10.2 | 0.733 | 8 |
| Nalidixic acid | J01MB02 | Unclassified | 7.9 | 1.5; 14.3 | 0.000 | 1 |
| Nitrofurantoin | J01XE01 | Access | 5.6 | 2.9; 8.2 | 0.661 | 2 |
| Doxycycline | J01AA02 | Access | 5.4 | 2.9; 8 | 0.745 | 3 |
| Norfloxacin | J01MA06 | Watch | 5 | 1.8; 8.2 | 0.252 | 2 |
| Phenoxymethylpenicillin | J01CE02 | Access | 3.9 | 2.3; 5.5 | 0.248 | 3 |
| Ofloxacin | J01MA02 | Watch | 3.6 | 0.7; 6.6 | 0.000 | 1 |

**Subgroup analyses using the Freeman-Tukey test**

**S2. Fig. 1.** Risk of Bias

**S2. Fig. 2.** Study design

**S2. Fig. 3.** Study period (year of data collection)

**S2. Fig. 4.** Type of patient

**S2. Fig. 5.** Geographic region

**S2. Fig. 6.** Sample size (Categorical)

**S2. Fig. 7.** Funnel plot of the Trim-and-fill method

**S2. Fig. 8.** Sensitivity analysis on the prevalence of antibiotic prescriptions

**Access Group**

**S2. Fig. 9.** Distribution of Access Groups by Region

**S2. Fig. 10.** Distribution of Access Groups by Country

**S2. Fig. 11.** Distribution of Access Groups by Patient

**Watch Group**

**S2. Fig. 12.** Distribution of Watch Groups by Region

**S2. Fig. 13.** Distribution of Watch Groups by Country

**S2. Fig. 14.** Distribution of Watch Groups by Patient

**Reserve Group**

**S2. Fig. 15.** Distribution of Reserve Groups by Region

**S2. Fig. 16.** Distribution of Reserve Groups by Country

**S2. Fig. 17.** Distribution of Reserve Groups by Patient

**Unclassified Group**

**S2. Fig. 18.** Distribution of Unclassified Groups by Region

**S2. Fig. 19.** Distribution of Unclassified Groups by Country

**S2. Fig. 20.** Distribution of Unclassified Groups by Patient

**Not recommended Group**

**S2. Fig. 21.** Distribution of Not recommended Groups by Region

**S2. Fig. 22.** Distribution of Not recommended Groups by Country

**S2. Fig. 23.** Distribution of Not recommended Groups by Patient

**S2. Table 2.** NIH Quality Assessment Tool for Observational Cohort and Cross-Sectional Studies

|  | **Author, Year** | **14 items into dichotomous variables: yes, no, or not applicable** | | | | | | | | | | | | | | **Total score** (from 14) | **Quaity rating**  (Good, Fair, or Poor) |
| --- | --- | --- | --- | --- | --- | --- | --- | --- | --- | --- | --- | --- | --- | --- | --- | --- | --- |
|  |  | 1 | 2 | 3 | 4 | 5 | 6 | 7 | 8 | 9 | 10 | 11 | 12 | 13 | 14 |  |  |
| 1 | Umeokonkwo et al., 2023 | 1 | 1 | 1 | 1 | 1 | NA | NA | 0 | 1 | NA | 1 | NA | NA | NA | 7 | Fair |
| 2 | Tack et al., 2023 | 1 | 1 | 1 | 1 | 1 | 0 | 1 | 1 | 1 | NA | 1 | NA | 1 | NA | 10 | Good |
| 3 | Tekleab et al., 2017 | 1 | 1 | 1 | 1 | 1 | NA | NA | 0 | 1 | NA | 1 | NA | NA | NA | 7 | Fair |
| 4 | Okoye et al., 2022 | 1 | 1 | 1 | 1 | 1 | NA | NA | 0 | 1 | NA | 1 | NA | NA | NA | 7 | Fair |
| 5 | Cole et al., 2015 | 1 | 1 | 1 | 1 | 1 | NA | NA | 0 | 1 | NA | 1 | NA | NA | NA | 7 | Fair |
| 6 | Sana et al., 2019 | 1 | 1 | 1 | 1 | 1 | NA | NA | 1 | 1 | NA | 1 | NA | NA | NA | 8 | Fair |
| 7 | Slogrove et al., 2010 | 1 | 1 | 1 | 1 | 1 | 1 | 1 | 0 | 1 | 0 | 1 | NA | 1 | NA | 9 | Fair |
| 8 | Salau et al., 2023 | 1 | 1 | 1 | 1 | 1 | NA | NA | 0 | 1 | NA | 1 | NA | NA | NA | 7 | Fair |
| 9 | Chaw et al., 2018 | 1 | 1 | 1 | 1 | 1 | NA | NA | 1 | 1 | NA | 1 | NA | NA | NA | 8 | Fair |
| 10 | Awuor et al., 2023 | 1 | 1 | 1 | 1 | 1 | NA | NA | 1 | 1 | 0 | 1 | NA | NA | NA | 8 | Fair |
| 11 | Kamara et al., 2024 | 1 | 1 | 1 | 1 | 1 | NA | NA | 0 | 1 | NA | 1 | NA | NA | NA | 7 | Fair |
| 12 | Olaru et al., 2020 | 1 | 1 | 1 | 1 | 1 | 1 | 1 | 0 | 1 | 0 | 1 | NA | 1 | NA | 10 | Good |
| 13 | Kitt et al., 2022 | 1 | 1 | 1 | 1 | 1 | 1 | 1 | 1 | 1 | 0 | 1 | NA | 1 | NA | 11 | Good |
| 14 | Merga et al., 2024 | 1 | 1 | 1 | 1 | 1 | NA | NA | 0 | 1 | NA | 1 | NA | 1 | NA | 8 | Fair |
| 15 | Akintan et al., 2024 | 1 | 1 | 1 | 1 | 1 | NA | NA | 0 | 1 | NA | 1 | NA | NA | NA | 7 | Fair |
| 16 | Obura et al., 2021 | 1 | 1 | 1 | 1 | 1 | 1 | 1 | 0 | 1 | 0 | 1 | NA | 1 | NA | 10 | Good |
| 17 | Skosana et al., 2022 | 1 | 1 | 1 | 1 | 1 | NA | NA | 0 | 1 | NA | 1 | NA | NA | NA | 7 | Fair |
| 18 | Ndayizeye et al., 2019 | 1 | 1 | 1 | 1 | 0 | NA | NA | 1 | 1 | NA | 1 | NA | NA | NA | 7 | Fair |
| 19 | Gres et al., 2024 | 1 | 1 | 1 | 1 | 1 | NA | NA | 1 | 1 | NA | 1 | NA | NA | NA | 8 | Fair |
| 20 | Xavier et al., 2024 | 1 | 1 | 1 | 1 | 1 | NA | NA | 1 | 1 | NA | 1 | NA | NA | NA | 8 | Fair |
| 21 | Gidey et al., 2024 | 1 | 1 | 1 | 1 | 1 | NA | NA | 1 | 1 | NA | 1 | NA | NA | NA | 8 | Fair |
| 22 | Mambula et al., 2023 | 1 | 1 | 1 | 1 | 1 | NA | NA | 0 | 1 | NA | 1 | NA | NA | NA | 7 | Fair |
| 23 | Tusubira et al., 2023 | 1 | 1 | 1 | 1 | 1 | NA | NA | 1 | 1 | NA | 1 | NA | NA | NA | 8 | Fair |
| 24 | Xavier et al., 2022 | 1 | 1 | 1 | 1 | 1 | NA | NA | 1 | 1 | NA | 1 | NA | NA | NA | 8 | Fair |
| 25 | Allwell-Brown et al., 2022 | 1 | 1 | 1 | 1 | 1 | NA | NA | 1 | 1 | NA | 1 | NA | NA | NA | 8 | Fair |
| 26 | Garedow and Tesfaye, 2022 | 1 | 1 | 1 | 1 | 1 | NA | NA | 1 | 1 | NA | 1 | NA | NA | NA | 8 | Fair |
| 27 | Alekaw et al., 2022 | 1 | 1 | 1 | 1 | 1 | NA | NA | 0 | 1 | NA | 1 | NA | NA | NA | 7 | Fair |
| 28 | Kakolwa et al., 2021 | 1 | 1 | 1 | 1 | 0 | NA | NA | 0 | 1 | NA | 1 | NA | NA | NA | 5 | Poor |
| 29 | Sié et al., 2021 | 1 | 1 | 1 | 1 | 0 | NA | NA | 0 | 1 | NA | 1 | NA | Na | NA | 6 | Fair |
| 30 | Yehualaw et al., 2021 | 1 | 1 | 1 | 1 | 1 | NA | NA | 0 | 1 | NA | 1 | NA | NA | NA | 7 | Fair |
| 31 | Garcia-Vello et al., 2020 | 1 | 1 | 1 | 1 | 0 | NA | NA | 0 | 1 | NA | 1 | NA | NA | NA | 6 | Fair |
| 32 | Sié et al., 2019 | 1 | 1 | 1 | 1 | 1 | NA | NA | 0 | 1 | NA | 1 | NA | NA | NA | 7 | Fair |
| 33 | Labi et al., 2018 | 1 | 1 | 1 | 1 | 1 | NA | NA | 0 | 1 | NA | 1 | NA | NA | NA | 7 | Fair |
| 34 | Morgan et al., 2018 | 1 | 1 | 1 | 1 | 1 | NA | NA | 1 | 1 | NA | 1 | NA | NA | NA | 8 | Fair |
| 35 | Adisa et al., 2018 | 1 | 1 | 1 | 1 | 1 | NA | NA | 0 | 1 | NA | 1 | NA | NA | NA | 7 | Fair |
| 36 | Umar et al., 2018 | 1 | 1 | 1 | 1 | 1 | NA | NA | 0 | 1 | NA | 1 | NA | NA | NA | 7 | Fair |
| 37 | Kebede et al., 2017 | 1 | 1 | 1 | 1 | 1 | NA | NA | 0 | 1 | NA | 0 | NA | NA | NA | 6 | Fair |
| 38 | Monteiro et al., 2017 | 1 | 1 | 1 | 1 | 0 | NA | NA | 0 | 1 | NA | 0 | NA | NA | NA | 5 | Poor |
| 39 | Koura et al., 2013 | 1 | 1 | 1 | 1 | 0 | NA | NA | 0 | 1 | NA | 1 | NA | NA | NA | 6 | Fair |
| 40 | Mabiala Babela et al., 2013 | 1 | 1 | 1 | 1 | 0 | NA | NA | 0 | 1 | NA | 1 | NA | NA | NA | 6 | Fair |
| 41 | Gwimile et al., 2012 | 1 | 1 | 1 | 1 | 1 | NA | NA | 0 | 1 | NA | 1 | NA | NA | NA | 7 | Fair |
| 42 | Rhee et al., 2019 | 1 | 1 | 1 | 1 | 1 | NA | NA | 0 | 1 | NA | 1 | NA | NA | NA | 7 | Fair |
| 43 | Pillay et al., 2024 | 1 | 1 | 1 | 1 | 1 | NA | NA | 0 | 1 | NA | 1 | NA | NA | NA | 7 | Fair |
