# Supplementary material for: National and regional challenges of antibiotic prescribing patterns in pediatric populations across Africa: a systematic review and meta-analysis
Source: Syst Rev. 2026 Jun 10;15:228. doi: 10.1186/s13643-026-03223-9 (PMC13480107; doi:10.1186/s13643-026-03223-9)
Supplement: Supplementary file 3 [file 13643_2026_3223_MOESM3_ESM.docx]

**Supplementary material (S3) - using DerSimonian–Laird random-effects**

**Fig. 1.** Forest plot of pooled patient-level prevalence of antibiotic prescribing in children in Africa

**Subgroup analysis**

**Fig. 2.** Risk of Bias

**Fig. 3.** Study design

**Fig. 4.** Study period (year of data collection)

**Fig. 5.** Type of patient

**Fig. 6.** Region

**Fig. 7.** Sample size (Categorical)

**Fig. 8.** Funnel plot of the Trim-and-fill method

**Fig. 9.** Sensitivity analysis on the prevalence of antibiotic prescriptions

**Access Group by region**

**Fig. 10.** Distribution of Access Groups by Region

**Fig. 11.** Distribution of Access Groups by Country

**Fig. 12.** Distribution of Access Groups by Patient

**Fig. 13.** Distribution of Watch Groups by Region

**Fig. 14.** Distribution of Watch Groups by Country

**Fig. 15.** Distribution of Watch Groups by Patient

**Reserve group**

**Fig. 16.** Distribution of Reserve Groups by Region

**Fig. 17.** Distribution of Reserve Groups by Country

**Fig. 18.** Distribution of Reserve Groups by Patient

**Unclassified group**

**Fig. 19.** Distribution of Unclassified Groups by Region

**Fig. 20.** Distribution of Unclassified Groups by Country

**Fig. 21.** Distribution of Unclassified Groups by Patient

**Not Recommended group**

**Fig. 22.** Distribution of Not recommended Groups by Region

**Fig. 23.** Distribution of Not recommended Groups by Country

**Fig. 24.** Distribution of Not recommended Groups by Patient
